# Impact of Asthma Phenotypes on Myocardial Performance and Pulmonary Hypertension in Children and Adolescents With Moderate to Severe Persistent Asthma

**DOI:** 10.7759/cureus.44252

**Published:** 2023-08-28

**Authors:** Rajkumar Kundavaram, Praveen Kumar, Shikha Malik, Girish Bhatt, Priya Gogia, Amber Kumar

**Affiliations:** 1 Pediatrics, All India Institute of Medical Sciences, Bhopal, Bhopal, IND

**Keywords:** diastolic dysfunction, wheeze, pediatric pulmonary hypertension, myocardial performance index, pediatric asthma

## Abstract

Background: Asthma is characterized by chronic inflammation and remodeling of pulmonary vessels and airway wall resulting in pulmonary hypertension (PH). Increased afterload on right ventricle (RV) myocardium leads to RV diastolic dysfunction (RVDD). Echocardiography is an excellent tool to detect these changes early. Using echocardiography, we assessed the impact of clinical asthma phenotypes on myocardial performance and PH in children with asthma.

Materials and methods: Sixty children with moderate or severe persistent asthma and 60 age and gender-matched healthy controls were enrolled. As per clinical phenotypes, children with asthma were classified into early wheezers (n = 30) and late wheezers (n = 30). Pulmonary function tests (PFT) and echocardiography, both conventional and pulse wave (PW), were performed.

Results: Children with asthma had significant RVDD and higher incidence (33%) of PH. Myocardial performance index (MPI) was poor in asthmatics, 0.41 (0.04) compared to controls, 0.38 (0.03). Measures for PH such as tricuspid regurgitation (TR) gradient, TR velocity, and pulmonary artery pressure (PAP) were significantly higher in cases.

Among clinical asthma phenotypes, there was no difference in left ventricular ejection fraction (LVEF) between early 64.3% (4.6) and late wheezers 65.6% (4.4). MPI was better in late wheezers at 0.41 (0.05) than in early wheezers at 0.40 (0.03). TR gradient, TR velocity, and PAP were significantly higher in early wheezers. The odds ratio for the development of PH was 0.74 (CI 0.25 - 2.17), and for the development of RVDD was 3.2 (CI 0.77 - 13.8), both in favor of early wheezers.

Conclusion: Children with asthma, particularly early-onset wheezers are at increased risk of developing PH and RVDD. We suggest annual screening by conventional echocardiography and pulse wave Doppler imaging for early diagnosis and timely initiation of management.

## Introduction

Asthma is a chronic airway inflammatory disorder characterized by various signs and symptoms like wheezing, cough, chest tightness, and respiratory distress [1]. These clinical features are due to airway hyperresponsiveness which results from environmental and genetic factors [2]. This chronic inflammation is not restricted only to the lungs but affects other organ systems, including the heart [3]. Pulmonary hypertension (PH) is a group of conditions with multiple causes rather than a single cause [4]. An increased pulmonary blood flow or vascular resistance can lead to pulmonary hypertension. Regardless of etiology, PH is characterized by anatomic changes in the pulmonary vasculature, further leading to structural and functional changes in the heart [3,5]. Asthmatic children are at increased risk of developing PH [3]. Multiple pathogenic mechanisms have been explained for pulmonary hypertension in patients with asthma.

Three major pathological features are common to asthma and PH: inflammation, smooth muscle constriction, and smooth muscle cell proliferation [5]. Recurrent hypoxia, hypercarbia, and various inflammatory mediators and cytokines are released due to chronic inflammation leading to pulmonary vasoconstriction [6]. Asthmatic airway inflammation causes tissue injury and related structural changes that may lead to airway remodeling in the form of an increase in airway wall thickness, fibrosis, smooth muscle mass and vascularity, and abnormalities in extracellular matrix composition [7].

Exaggerated breathing efforts in patients with asthma increase the intrathoracic pressure thereby increasing the right ventricular afterload [8]. Because of chronic pressure overload, the right ventricle (RV) hypertrophies and dilates, resulting in systolic and diastolic dysfunction [9]. Tissue Doppler and pulse wave (PW) Doppler imaging have emerged as one of the most potent prognosticators for cardiovascular function within the spectrum of non-invasive cardiac imaging [10]. To our knowledge, there are scant Indian studies that measured pulmonary hypertension and right ventricle myocardial performance index (MPI) using pulsed wave Doppler imaging in asthmatic children. Tissue Doppler imaging (TDI) and pulse wave Doppler have emerged as non-invasive diagnostic modalities to assess cardiac functions and to detect subclinical myocardial dysfunctions in different clinical studies, including obesity, cytotoxic-induced cardiomyopathy, congenital heart diseases and pulmonary hypertension [11,12]. Also, it has good repeatability and reproducibility, as reported in many studies like Puleo et al. [13], Daniel et al. [14], and Cui et al. [15].

## Materials and methods

A single-centre, cross-sectional study was conducted in the Department of Paediatrics at a tertiary care hospital in central India. Institutional human ethics committee approval was taken before the study (approval IHECPGRMD041). Informed consent for the study was obtained from the parents of the patients. It was a time bound study based on operational feasibility. The study was performed on 60 children and adolescents (age group six to 18 years) with moderate and severe persistent asthma who could perform spirometry. Out of which, we enrolled 30 early-onset wheezer phenotypes (onset of wheezing in first three years of life and persistent through six years of life) and 30 late-onset wheezer phenotypes (onset of wheezing between three and six years of age). We also enrolled 30 age- and sex-matched healthy controls for each group. For all enrolled participants, a detailed history was taken with particular reference to the patient’s age, age at diagnosis, severity of asthma, frequency of exacerbation, history of oxygen requirement and hospital admission was noted in a predesigned proforma. Spirometry and conventional and pulse wave Doppler echocardiography were performed in all these asthma cases in well-controlled status and were noted in a predesigned proforma.

Spirometry parameters were recorded: forced expiratory volume in initial one second (FEV1), functional vital capacity (FVC) and their ratio FEV1/FVC were recorded for all enrolled children. FEV1 is the air exhaled in an initial one second during forced exhalation in spirometry. FVC is the maximal amount of air that can be expelled from the lungs after a maximal inspiratory effort. FVC and FEV1 results above 80% of the predicted mean value were considered within the normal range [16]. FEV1/FVC ratio above 80% was considered normal and decreased to <80% in obstructive lung disease because airflow is reduced in obstructive lung conditions.

An echocardiographic assessment of myocardial performance and pulmonary hypertension was performed using an EPIQ 7 colour Doppler ultrasound machine (Philips, Amsterdam, the Netherlands). All echocardiograms were performed by a paediatric cardiologist, who was blinded regarding the patient's clinical diagnosis. Structural heart disease was ruled out initially. MPI is a numeric value that can be obtained by adding the isovolumetric relaxation time (IRT) and isovolumetric contraction time (ICT) of each ventricle. Pulse Doppler echocardiography was done to calculate the MPI of the right ventricle, a value greater than 0.43 is considered abnormal [12,17].

Pulmonary artery systolic pressure quantification was done with the help of tricuspid regurgitation (TR) velocity (TRV). TR was assessed with parasternal short-axis, parasternal long-axis right ventricular inflow and apical four-chamber to determine a good quality velocity/time wave and the highest velocity obtained from any of the three views was selected as the peak TRV (m/s). TRV was used to estimate the pulmonary artery systolic pressure using the Bernoulli equation (pulmonary artery systolic pressure = 4 (TRV)2 + right atrial pressure). Pulmonary hypertension was defined as a peak TRV of at least 2.5 m/second, equating to a pulmonary artery pressure of at least 30 mmHg [18]. Mild pulmonary hypertension is a peak TRV of 2.5 to 2.9m/second, corresponding to pulmonary artery systolic pressure of 30 to 39 mmHg. Moderate pulmonary hypertension is a peak TRV ≥ 3 m/second corresponding to a pulmonary systolic pressure of 40 to 70 mmHg, and severe pulmonary hypertension corresponds to pulmonary artery systolic pressure of >70 mmHg. Patients with no measurable TRV or TRV <2.5 m/second were considered to have normal pulmonary artery pressures and normal tricuspid flow.

Quantification of pulmonary artery acceleration time on pulse Doppler tracings across the pulmonary artery was also assessed. There were situations where no tricuspid regurgitation was present, or the signal was inadequate to obtain reliable measurements. In these cases, looking at the PW Doppler signal of pulmonary flow was precious. The normal spectrum is more or less symmetrical in shape. Its maximum velocity is in the range of 0.8 -1.2 m/sec. The peak will occur earlier when pulmonary artery pressure (PAP) and vascular resistance are high. This was quantified using the pulmonary acceleration time (PAT). It is the interval between the onset of flow and peak flow. The normal PAT is > 130 msec. Using pulmonary acceleration time and the systolic notch, the severity of pulmonary hypertension was estimated and graded as mild elevation (80-100 milli sec) and severely elevated PAP (less than 80 milli sec or systolic notch).

## Results

Microsoft Excel (Microsoft, Redmond, WA, USA) for data entry and EPI Info 7 (CDC, Atlanta, GA, USA), SPSS Statistics v.23 (IBM Corp., Armonk, NY, USA) were used for data analysis. A total of 60 asthmatic patients, 11.5 years (3.2), and 60 healthy controls, 10.8 years (2.8), were included in the study. The demographic parameters, clinical features, and laboratory values of healthy controls and patients with asthma are shown in Table 1.

**Table 1:** Demographic variables, clinical features, and laboratory values of study participants

Variable	Controls ( N=60)	Cases (N=60)	P
Age (years)	11.6 (3.2)	10.9 (2.9)	0.205
Male	31	34	
Female	29	26	
Body weight (kg)	33 (8.9)	32.4 (9.3)	0.733
Height (cm)	131 (16)	130 (17)	0.806
Body Mass Index (kg/m2)	20 (7)	19.1 (4)	0.354
Heart rate (beats/min)	95 (12)	95 (12)	0.876
Systolic Blood Pressure (mm Hg)	106 (9)	106 (9)	0.968
Diastolic Blood Pressure (mm Hg)	66 (7)	66 (7)	0.938
Hemoglobin (gm%)	12.7 (1.4)	12.6 (1.5)	0.709
Platelets (lakhs/mm3)	2.7 (0.9)	2.7 (0.9)	0.943
Total Leucocyte Count (thousands/mm3)	7.6 (1.8)	7.7 (2.0)	0.673
Absolute Eosinophil Count (/mm3)	559 (270)	657 (398)	0.117

No statistically significant differences were found between the patients and controls regarding age, sex, height, weight, BMI, heart rate (HR), systolic and diastolic blood pressures, and total eosinophil counts. As expected, healthy controls have better spirometry parameters (FEV1/FVC) as compared to patients with asthma (71.8 and 79.2, respectively), and this is statistically significant (p - 0.0000) (Table 2). On assessing echocardiography parameters between cases and controls, no difference was found in left ventricle ejection fraction (64.3% and 65%). MPI was better in controls at 0.38 (0.03) than in cases at 0.41 (0.04). All the measures for pulmonary hypertension, such as TR gradient, TR velocity, and PAP, were significantly higher in cases (Table 2).

**Table 2:** Spirometry and echocardiography parameters of study participants FEV1: forced expiratory volume in initial one second FVC: functional vital capacity EF: ejection fraction MPI: myocardial performance index TR: tricuspid regurgitation PAT: pulmonary acceleration time PAP: pulmonary artery pressure

Parameter	Controls (N=60)	Cases (N=60)	P
FEV1 (litre)	1.14 (0.2)	1.03 (0.3)	0.0258
FVC (litre)	1.43 (0.3)	1.43 (0.3)	0.9487
FEV1/FVC (%)	71.8 (8.9)	79.2 (6.7)	0.0000
EF (%)	64.3 (8.3)	65 (4.6)	0.595
MPI	0.38 (0.03)	0.41 (0.04)	0.0009
TR gradient (mmHg)	17.1 (3.5)	21 (7.1)	0.0003
TR velocity (m/sec)	2 (0.2)	2.3 (0.4)	0.0003
PAT (milli sec)	152.3 (10.6)	136.7 (16.8)	0.0000
PAP Systolic (mmHg)	26 (2)	31 (9)	0.0000
PAP diastolic(mmHg)	9.5 (2)	10.5 (4)	0.0779
PAP mean(mmHg)	16 (2)	19 (7)	0.0013

**Figure 1:**
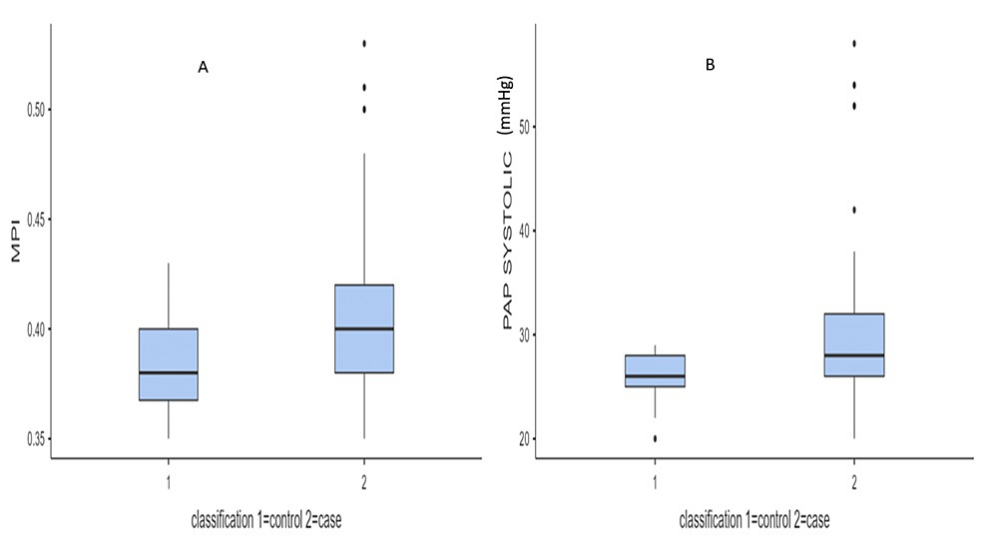
Box whisker plot showing myocardial performance index (MPI) and systolic pulmonary artery pressure (PAP) in controls versus cases

Cases and controls were analysed for pulmonary hypertension and myocardial dysfunction. There was no pulmonary hypertension or myocardial dysfunction in the controls. Among cases, 20 patients (33%) had pulmonary hypertension, and 11 (18.3%) had right ventricular diastolic dysfunction. MPI and systolic PAP among cases and controls are shown in Figure 1.

Among cases, clinical phenotype groups of early versus late wheezers were also compared (Table 3). There was no statistical difference in demographic variables between 30 early wheezers at 11.2 years (3.1) and 30 late wheezers at 10.5 years (2.6). Spirometry parameters FEV1/FVC was better in early wheezers 78.3 (6.9) than late wheezers 65.3 (5.3). There was no effect of inhaled steroid use on lung function; however, only a small percentage of late wheezers (50%) reported the use of this medication compared with early wheezers (80%), and we did not characterize dose or duration, thus limiting the applicability of this negative finding. Further findings of the Childhood Asthma Management Program study showed that school children with asthma had relatively stable levels of lung function during a four- to six-year follow-up, even when they were not treated with inhaled anti-inflammatory medication. There was no difference in left ventricular ejection fraction between early 64.3% (4.6) and late wheezers 65.6 (4.4). MPI was better in late wheezers 0.41 (0.05) than in early wheezers 0.40 (0.03). Measures of pulmonary hypertension such as TR gradient, TR velocity, and PAP were significantly higher in early wheezers (Table 3).

**Table 3:** Comparison of early versus late wheezers FEV1: forced expiratory volume in initial one second FVC: functional vital capacity EF: ejection fraction MPI: myocardial performance index TR: tricuspid regurgitation PAT: pulmonary acceleration time PAP: pulmonary artery pressure

Parameter	Early Wheezers (N=30)	Late Wheezers (N=30)	P
Age (years)	11.2 (3.1)	10.5 (2.6)	0.16
Male	63%	50%	-
Female	37%	50%	-
FEV1 (litre)	1.08 (0.34)	0.98 (0.25)	0.04
FVC (litre)	1.40 (0.42)	1.47 (0.32)	0.467
FEV1/FVC (%)	78.3 (6.9)	65.3 (5.3)	0.000
EF (%)	64.3 (4.6)	65.6 (4.4)	0.241
MPI	0.40 (0.01)	0.41 (0.05)	0.020
TR gradient (mmHg)	22.2 (6.9)	19.6 (7.1)	0.013
TR velocity (m/sec)	2.3 (0.38)	2.2 (0.45)	0.011
PAT (milli sec)	134.2 (18)	139.2 (15.4)	0.0004
PAP Systolic (mmHg)	31.9 (9.7)	30.3 (8.6)	0.02
PAP Diastolic (mmHg)	10.3 (3.6)	10.6 (4.1)	0.07
PAP Mean (mmHg)	18.6 (7.9)	19 (5.8)	0.31

**Figure 2:**
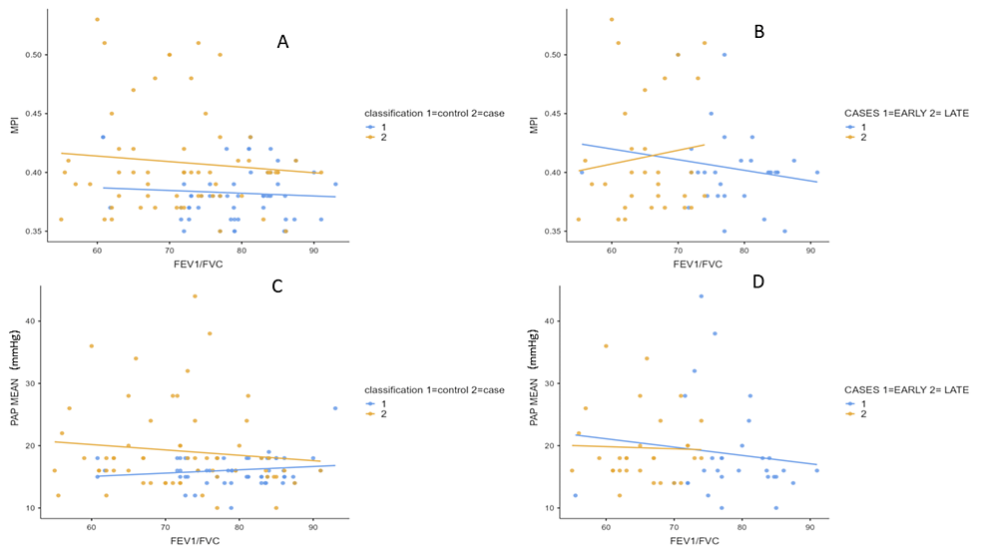
Scatter plot depicting FEV1/FVC versus MPI and mean PAP A: FEV1/FVC vs MPI cases and controls, B: FEV1/FVC vs mean PAP cases and controls, C: FEV1/FVC vs MPI early and late wheezers, D: FEV1/FVC vs mean PAP early and late wheezers FEV1: forced expiratory volume in initial one second FVC: functional vital capacity MPI: myocardial performance index PAP: pulmonary artery pressure

The odds ratio for the development of PH was 0.74 (CI 0.25 - 2.17) in favour of early wheezers. The odds ratio for the development of right ventricular diastolic dysfunction was 3.2 (CI 0.77 - 13.8) in favour of early wheezers. A positive correlation was found between FEV1 with TR gradient and mean PAP, FVC with TR gradient, systolic, and mean PAP. A negative correlation was observed between FEV1/FVC and MPI, mean PAP among early wheezers (Table 4, Figure 2).

**Table 4:** Correlation of FEV1/FVC versus MPI and PAP mean among all cases and early wheezers FEV1: forced expiratory volume in initial one second FVC: functional vital capacity MPI: myocardial performance index PAP: pulmonary artery pressure

	All cases		Early wheezers	
	FEV1/FVC vs MPI	FEV1/FVC vs PAP mean	FEV1/FVC vs MPI	FEV1/FVC vs PAP mean
Pearsons r	-0.097	-0.112	-0.183	-0.116
Df	58	58	28	28
P value	0.463	0.396	0.334	0.540
95% CI Upper	0.161	0.146	0.190	0.255
95% CI Lower	-0.342	-0.356	-0.509	-0.457

## Discussion

The present study results show that children and adolescents with moderate to severe persistent asthma have an increased incidence of pulmonary hypertension and right ventricular dysfunction compared to healthy controls. Left ventricular (LV) systolic function (ejection fraction) was similar in both cases and controls.

Chronic inflammation and pulmonary remodelling lead to an increased incidence of pulmonary hypertension in patients with asthma [15]. Pulmonary hypertension causes changes in ventricle structure and function due to increased afterload imposed on the myocardium of the right ventricle [3,5]. Right ventricle diastolic function is the earliest hemodynamic change in asthmatic children [10,19,20].

In our study we found the incidence of pulmonary hypertension to be 33%; we couldn’t find any studies on asthmatic children reporting the incidence of pulmonary hypertension. The incidence of right ventricular dysfunction was 11%. This was far less than 56.2% in a study by Wagdey et al. [16]. In this study, MPI in controls is 0.38 ±0.03, and in the cases is 0.41 ±0.04. Ozdemir et al. [21] conducted a study among 51 children with asthma and 46 age and gender-matched controls. MPI in asthmatics was 0.48 ± 0.07 compared to 0.42 ± 0.06 in the control group. De-Paula et al. [13], in their study among a total of 18 healthy controls and 20 asthmatic children and adolescents, found statistically significant (p=0.038) elevation in MPI with mean MPI of 0.40 ± 0.01 among controls (n=18) as compared to 0.43 ± 0.01 among asthmatic group (n=20). Ozde et al. [21], in a study on 68 paediatric patients with asthma and 69 age- and gender-matched healthy children, RV MPI was significantly higher in the asthma group (mean MPI = 0.28 ± 0.06) than in the control group (mean MPI = 0.24 ± 0.07) (p-value = 0.003). There is no similar study on Indian children for comparison.

Myocardial performance can be affected differently depending on the clinical presentation of asthma [20]. Children with asthma who prefer shallow breathing have more severe cardiac dysfunction than those who prefer wheezing as a symptom [21]. Very few studies evaluated myocardial performance in phenotypes of children with asthma and their response to medications. Ozdemir et al. [20] and Zedan et al. [22] utilised echocardiography in asthmatic patients and opined that pathogenesis, systemic effects and response to medication is different in different phenotypes. Some studies have evaluated myocardial changes in asthma children using tissue Doppler imaging but a comparison of clinical phenotypes of asthma was not done [23,24]. The unique feature of our study is the evaluation of cardiac performance by conventional and pulse wave Doppler in asthmatic patients according to their clinical phenotypes (early versus late wheezers).

MPI was better in late wheezers 0.41 (0.05) than in early wheezers 0.40 (0.03). Measures for pulmonary hypertension, such as TR gradient, TR velocity and PAP, were significantly higher in early wheezers. Also, the odds ratio for the development of RV diastolic dysfunction (RVDD) was 3.2 (CI 0.77 - 13.8) in favour of early wheezers. This supports that children with early onset wheezing are more prone to develop right ventricular dysfunction.

A positive correlation was found between FEV1 with TR gradient and mean PAP, FVC with TR gradient, systolic, and mean PAP. This supports indirect evidence that poor spirometry parameters are associated with an increased incidence of pulmonary hypertension. This is further substantiated by the fact that the odds ratio for the development of PH was 0.74 (CI 0.25 - 2.17) in favour of early wheezers.

Limitations of this study, apart from the small sample size localised from central India, was that the cases with pulmonary hypertension were not confirmed on cardiac catheterisation, which is considered to be the gold standard. However, the assessment of ventricular function by pulse wave echocardiography is well-studied and validated, and we demonstrated the role of echocardiography in children with asthma in the present study.

## Conclusions

In general, children with asthma and early-onset wheezers are at increased risk of developing PH and right ventricular dysfunction. Both PH and RVDD can easily be assessed by conventional echocardiography and pulse wave Doppler imaging. Parents of children with asthma and clinicians treating them should be aware of the risk of cardiac dysfunction and the need for timely follow-up. Early screening by echocardiography should be employed to detect damage and initiate management. More extensive prospective studies are recommended to evaluate further the impact of clinical asthma phenotypes on myocardial dysfunction. We suggest annual screening of children with asthma by conventional echocardiography and pulse wave Doppler imaging.
